# Atherogenic Index of Plasma as a Mediator in the association between Body Roundness Index and Depression: insights from NHANES 2005–2018

**DOI:** 10.1186/s12944-024-02177-y

**Published:** 2024-06-12

**Authors:** Genshan Zhang, Haokun Zhang, Jie Fu, Yufeng Zhao

**Affiliations:** 1 Department of Vascular Surgery, First Hospital of Lanzhou University, Lanzhou, 730030 China; 2 Department of Gastrointestinal Surgery, Tongji Hospital, Tongji Medical College, Huazhong University of Science and Technology, Wuhan, 430030 PR China; 3 School of Public Health and Health Management, Gannan Medical University, Ganzhou, 341000 PR China; 4 Department of Nursing, Tongji Hospital, Tongji Medical College, Huazhong University of Science and Technology, Wuhan, 430030 PR China

**Keywords:** Body roundness index, Visceral obesity, Depression, Dyslipidaemia, National Health and Nutrition Examination Survey

## Abstract

**Background:**

Previous studies have shown a correlation between depression and obesity, as well as between depression and the Atherogenic Index of Plasma (AIP). However, there is limited research on the association between visceral obesity and depression, as well as the potential mediating role of AIP in this relationship.

**Methods:**

This study included 13,123 participants from the 2005–2018 National Health and Nutrition Examination Survey. Visceral obesity was measured with the Body Roundness Index (BRI), while depression was evaluated with the Patient Health Questionnaire-9. The AIP served as a marker for lipid disorders. To investigate the association between the BRI and depression, multivariate logistic regressions, restricted cubic spline models, subgroup analyses, and interaction tests were used. Additionally, a mediation analysis was conducted to explore the role of AIP in mediating the effect of BRI on depression.

**Results:**

There was a positive linear correlation between the BRI and depression. After controlling for all covariates, individuals in the highest BRI (Q4) group had an OR of 1.42 for depression (95% CI: 1.12–1.82) in comparison with individuals in the lowest BRI (Q1) group. Moreover, the AIP partially mediated the association between the BRI and depression, accounting for approximately 8.64% (95% CI: 2.04-16.00%) of the total effect.

**Conclusion:**

The BRI was positively associated with depression, with the AIP playing a mediating role. This study provides a novel perspective on the mechanism that connects visceral obesity to depression. Managing visceral fat and monitoring AIP levels may contribute to alleviating depression.

**Supplementary Information:**

The online version contains supplementary material available at 10.1186/s12944-024-02177-y.

## Introduction

Depression is a prevalent psychological disorder marked by feelings of sadness and diminished interest in activities [1, 2]. This condition damages individuals’ physical, mental, and social well-being, exacerbating the burden on public health [3]. Previous researches have shown a greater prevalence of depression among obese individuals [4]. However, Body Mass Index (BMI) was deemed as the major indicator to assess obesity in these studies. While BMI can effectively evaluate the relationship between overall obesity and diseases [5], it does not effectively reflect the impact of fat distribution on depression. Additionally, waist circumference (WC), an indicator used to assess abdominal obesity, cannot accurately differentiate between subcutaneous fat and visceral fat [6]. Visceral fat is considered more harmful than fat in other parts of the body, and even individuals with a normal BMI or WC may have a significant accumulation of visceral fat [7]. Thomas and his team introduced the Body Roundness Index (BRI) through mathematical modeling to assess visceral fat levels [8]. Compared to WC, the BRI offers a more accurate depiction of visceral fat distribution [8]. Moreover, the BRI has been proven to be a convenient, fast, and cost-efficient alternative to approaches requiring X-ray scans for visceral fat evaluation [9]. Previous studies have shown correlations between the BRI and various health conditions, such as diabetes [10], and cardiovascular diseases (CVDs) [11]. However, there is limited research on the relationship between the BRI and depression.

Previous studies have identified dyslipidemia as a contributing factor to the occurrence and progression of depression [12, 13]. A newly recognized lipid marker named the atherogenic index of plasma (AIP) is used to evaluate lipid metabolism disorders [14]. The AIP was originally used for the prediction of atherosclerosis risk [15]. However, recent studies have shown that there was an association between the AIP and the incidence of depression [16–18]. Furthermore, previous studies have indicated strong correlations between visceral obesity and dyslipidemia [19, 20]. Considering that lipids and AIP can serve as indicators for drug intervention, and can help differentiate the risk of depression among patients with visceral obesity, investigating the mediating effect of AIP on the relationship between BRI and depression is of significant importance. Therefore, in this study, we utilized a large dataset from the National Health and Nutrition Examination Survey (NHANES) to investigate the relationship between BRI and depression. Additionally, we hypothesized that BRI could be associated with AIP, and that AIP might serve as a mediator between BRI and depression. To reveal this mediating effect, a two-step mediation analysis was employed.

## Methods

### Data source and participants selection

NHANES is a project of the National Center for Health Statistics that provides a thorough and continuous assessment of the health and nutrition of the American population. A sophisticated stratified sampling methodology was applied in the NHANES to enhance the accuracy and reliability of representative samples. Comprehensive data, including socioeconomic status, demographic characteristics, dietary habits, and health-related information, are collected by trained personnel. All participants must provide signed consent forms in order to participate in the study.

**Fig. 1:**
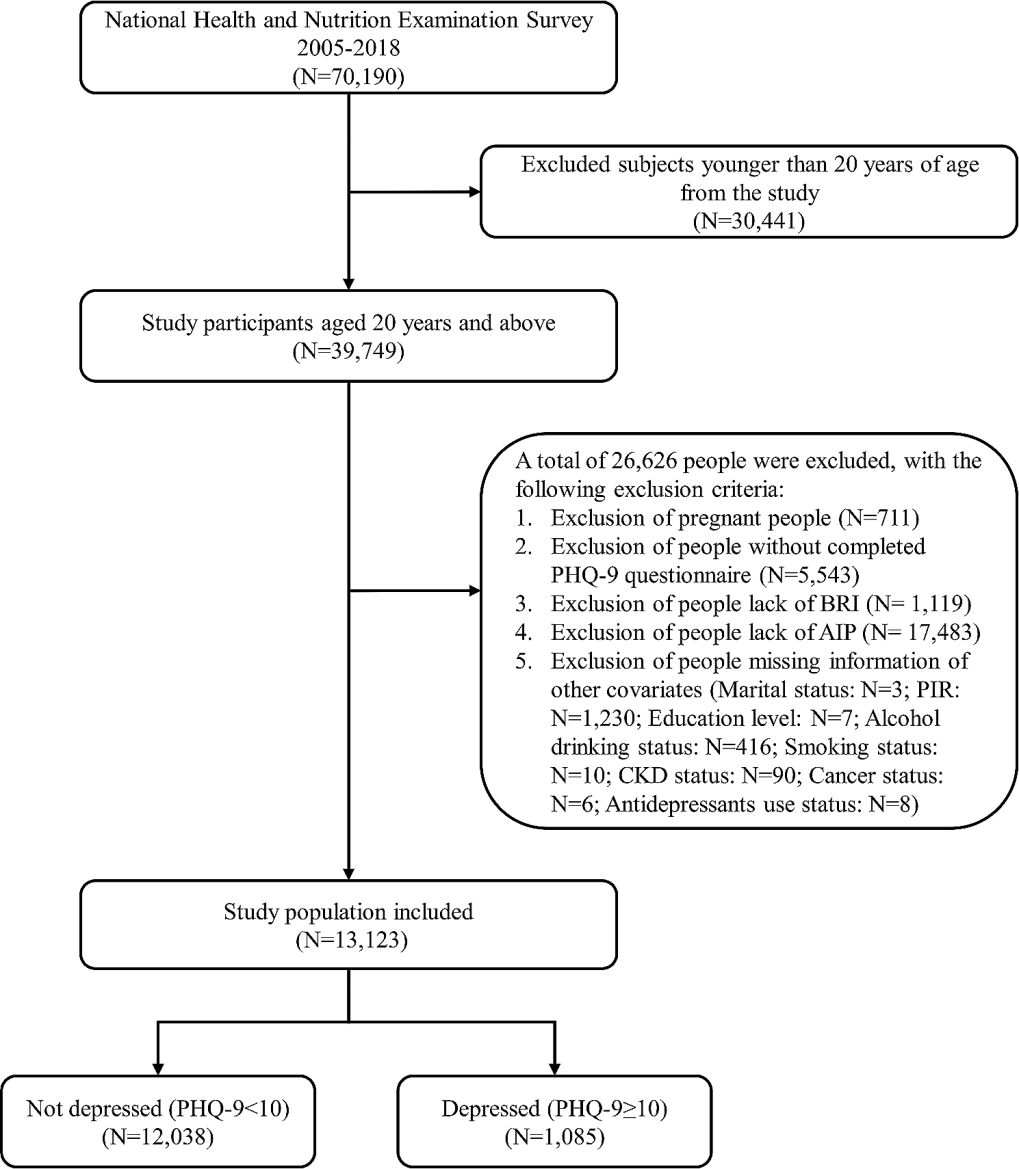
Flowchart of the sample selection from NHANES 2005–2018

This study utilized data from the NHANES cycles from 2005 to 2018, aligning with the availability of Patient Health Questionnaire-9 (PHQ-9) from 2005 to 2018. The present investigation included a total of 70,190 participants from these cycles. In the analysis, 30,441 participants under the age of 20 years were excluded. Additionally, 771 pregnant individuals were excluded due to alterations in blood lipid profiles, WC, and depression status. Those with missing PHQ-9 data (*n* = 5,543), BRI data (*n* = 1,119), and AIP data (*n* = 17,483) were also omitted. Individuals without information on covariates such as alcohol consumption status, smoking status, poverty income ratio (PIR), marital status, and education level were excluded (*n* = 1,770) (Fig. 1). Ultimately, this study included 13,123 individuals.

### Ascertainment of depression

The PHQ-9 [21] is a questionnaire widely used for screening depression. It consists of a total of 9 questions, graded on criteria from 0 to 3, resulting in a cumulative score scale from 0 to 27. A total score of 10 or higher was indicative of the presence of depression [21]. This cut-off point is commonly used in epidemiological research for the identification of individuals with depression and has been validated through clinical assessment [21].

### Ascertainment of the BRI

The model proposed by Thomas et al. was used to calculate the BRI [8]. This model incorporates two primary variables (height, and WC), to evaluate visceral fat content. A higher BRI indicates a greater accumulation of visceral fat. The specific mathematical formula for BRI calculation is as follows: 364.2-365.5 × (1-[WC (m)/2π]^2^/[0.5×height(m)]^2^)^½^. The BRI is categorized into four levels, ranging from low to high with the quartile intervals as follows: Q1 (1.19 ~ 3.82), Q2 (3.82 ~ 5.07), Q3 (5.07 ~ 6.67), and Q4 (6.67 ~ 19.00).

### Ascertainment of the AIP

The calculation of the AIP is based on indicators of high-density lipoprotein cholesterol (HDL-C) and triglyceride (TG) levels in the blood. The specific mathematical formula for AIP calculation is as follows: log10 [TG (mmol/L)/HDL-C (mmol/L)] [14].

### Covariates

In this study, the covariates included demographic characteristics (age, sex, and race), socioeconomic indicators (marital status, PIR, and education level), alcohol consumption status, smoking status, antidepressant use, and health conditions. Marital status was categorized as coupled (including married or living with a partner) and single/separated (including never married, separated, divorced, or widowed). Race was classified as non-Hispanic Black, non-Hispanic Asian, non-Hispanic White, other Hispanic (including Mexican American), or other. The participants’ education level was divided into three levels: less than high school, high school, and above high school. The PIR was grouped into three categories: < 1.30, 1.31 ~ 3.50, and > 3.50, with a higher PIR reflecting a better family economic status [22, 23]. Alcohol consumption condition was categorized into three groups: never drinkers (those who had consumed < 12 times in their lifetime), former drinkers (those who had consumed ≥ 12 times in a year but had not consumed any alcohol in the past year or did not consume alcohol in the last year but had consumed ≥ 12 times in their lifetime), and current drinkers (those who currently consumed at least one drink) [24, 25]. Detailed information on smoking status, diabetes status, CVD status, chronic kidney disease (CKD) status, cancer status, and antidepressant use is provided in Table S1.

### Statistical analysis

To enhance the representativeness of the research results, we followed the NHANES official recommended weighted procedures to process the data in this study. Based on the PHQ-9 scores of the participants, this study classified them into two groups: depression and non-depression [21]. Statistical analysis was conducted using Student *t* tests to compare the continuous variables and chi-square tests to compare the categorical variables between the two groups. To explore the relationship between the BRI and depression, weighted linear regression models (for continuous PHQ-9 scores) and logistic regression models (for depression) were used in the three statistical models to estimate the 95% confidence intervals (CIs) and adjusted odds ratios (ORs). Model 1 served as a crude model with no adjustments of variables. Model 2 was adjusted for sociodemographic factors (age, sex, and race) [26]. Model 3 was more adjusted for the PIR, marital status, education level, alcohol consumption status, smoking status, CVD status, diabetes status, CKD status, cancer status, and antidepressant use. In these models, when the BRI was considered an ordered four-category variable, trend tests were also conducted. Additionally, restricted cubic spline (RCS) analysis was conducted to determine whether the association between BRI and depression is linear. We also conducted subgroup analyses to assess the influence of the BRI on depression concerning several stratified covariates, including age (category), sex, PIR, education level, and disease status (diabetes, CVD, CKD, and cancer).

The two-step mediation analysis was used to evaluate the mediating effect of AIP. Firstly, a fully adjusted regression model was employed to investigate the impact of the BRI on AIP as well as the impact of the AIP on depression, aiming to ascertain the potential of the AIP to serve as a mediating factor between the BRI and depression. Subsequently, mediation analysis was conducted using the RMediation package to assess the indirect, direct, and overall effect of the BRI on depression mediated by the AIP [27]. After dividing the indirect effect by the total effect, the percentage of the mediating effect mediated by the AIP was determined. The 95% CI for the mediated proportion was estimated through nonparametric bootstrapping with 1000 iterations.

All statistical analyses were conducted using the R software (version 4.2.3). When the two-sided *P* value ≤ 0.05, it is considered statistically significant.

## Results

### Basic information

As shown in Table 1, this study comprised 13,123 individuals with a mean age of 47.51 ± 0.55 years. Of all these individuals, 1,085 (8.27%) were identified as having depression (PHQ-9 score ≥ 10). Females and individuals with a single/separated marital status exhibited a greater prevalence of depression. Moreover, individuals with depression tended to have lower education and income levels. Furthermore, the BRI of individuals with depression was higher than those without depression (*P* < 0.001).

**Table 1:** Weighted characteristics of the study population based on depression

Characteristic	Total	PHQ-9 < 10	PHQ-9 ≥ 10	*P* value
Age (years)	47.51 (46.96, 48.06)	47.51 (46.93, 48.09)	47.49 (46.33, 48.66)	0.98
Age group (year), n (%)				0.09
< 60	8776 (74.27)	8013 (74.07)	763 (76.92)	
≥ 60	4347 (25.73)	4025 (25.93)	322 (23.08)	
Sex (%)				< 0.001
Male	6612 (49.95)	6212 (50.91)	400 (37.56)	
Female	6511 (50.05)	5826 (49.09)	685 (62.44)	
Race (%)				0.04
Non-Hispanic White	2913 (39.67)	2643 (39.56)	270 (41.18)	
Non-Hispanic Asian	847 (2.71)	821 (2.82)	26 (1.31)	
Non-Hispanic Black	1460 (5.46)	1339 (5.37)	121 (6.64)	
Other Hispanic	1712 (8.10)	1553 (8.02)	159 (9.15)	
Others	6191 (44.05)	5682 (44.23)	509 (41.73)	
Education level (%)				< 0.001
Less than high school	1210 (4.81)	1060 (4.52)	150 (8.52)	
High school	4853 (33.31)	4355 (32.51)	498 (43.73)	
More than high school	7060 (61.88)	6623 (62.97)	437 (47.74)	
Marital, N (%)				< 0.001
Coupled	7961 (64.70)	7478 (65.94)	483 (48.63)	
Single or separated	5162 (35.30)	4560 (34.06)	602 (51.37)	
Poverty ratio				< 0.001
< 1.30	3978 (20.14)	3391 (18.43)	587 (42.38)	
1.31 ~ 3.50	5025 (36.22)	4683 (36.39)	342 (34.00)	
> 3.50	4120 (43.64)	3964 (45.18)	156 (23.62)	
Alcohol drinking status				< 0.001
Never	1720 (10.13)	1574 (10.17)	146 (9.63)	
Former	2125 (13.24)	1910 (12.85)	215 (18.35)	
Now	9278 (76.63)	8554 (76.99)	724 (72.02)	
Smoking status				< 0.001
Never	7110 (54.11)	6678 (55.49)	432 (36.27)	
Former	3285 (25.77)	3039 (25.98)	246 (23.14)	
Now	2728 (20.11)	2321 (18.53)	407 (40.59)	
CVD (%)				< 0.001
No	11,715 (91.29)	10,848 (91.84)	867 (84.11)	
Yes	1408 (8.71)	1190 (8.16)	218 (15.89)	
Diabetes (%)				< 0.001
No	10,399 (84.26)	9619 (84.75)	780 (77.93)	
Yes	2724 (15.74)	2419 (15.25)	305 (22.07)	
Cancer (%)				0.41
No	11,923 (90.50)	10,952 (90.58)	971 (89.45)	
Yes	1200 (9.50)	1086 (9.42)	114 (10.55)	
CKD (%)				< 0.001
No	10,850 (86.61)	9996 (86.90)	854 (82.88)	
Yes	2273 (13.39)	2042 (13.10)	231 (17.12)	
Antidepressants use (%)				< 0.001
No	11,644 (86.22)	10,931 (88.26)	713 (59.75)	
Yes	1479 (13.78)	1107 (11.74)	372 (40.25)	
Triglyceride (mg/dl)	126.72 (123.94, 129.49)	125.58 (122.82, 128.34)	141.50 (133.31, 149.68)	< 0.001
HDL cholesterol (mg/dl)	54.30 (53.81, 54.78)	54.41 (53.92, 54.91)	52.79 (51.55, 54.03)	0.01
AIP	-0.06 (-0.07, -0.05)	-0.06 (-0.07, -0.05)	0.00 (-0.02, 0.03)	< 0.001
BRI	5.34 (5.27, 5.42)	5.29 (5.22, 5.36)	6.03 (5.84, 6.22)	< 0.001

Mean ± SD for continuous variables: *P*-value was calculated by weighted linear regression model. (%) for categorical variables: *P* value was calculated by weighted chi-square test

CVD, cardiovascular disease; CKD, chronic kidney disease; AIP, Plasma atherogenic index; BRI, body roundness index

### Relationship between the BRI and depression

**Fig. 2:**
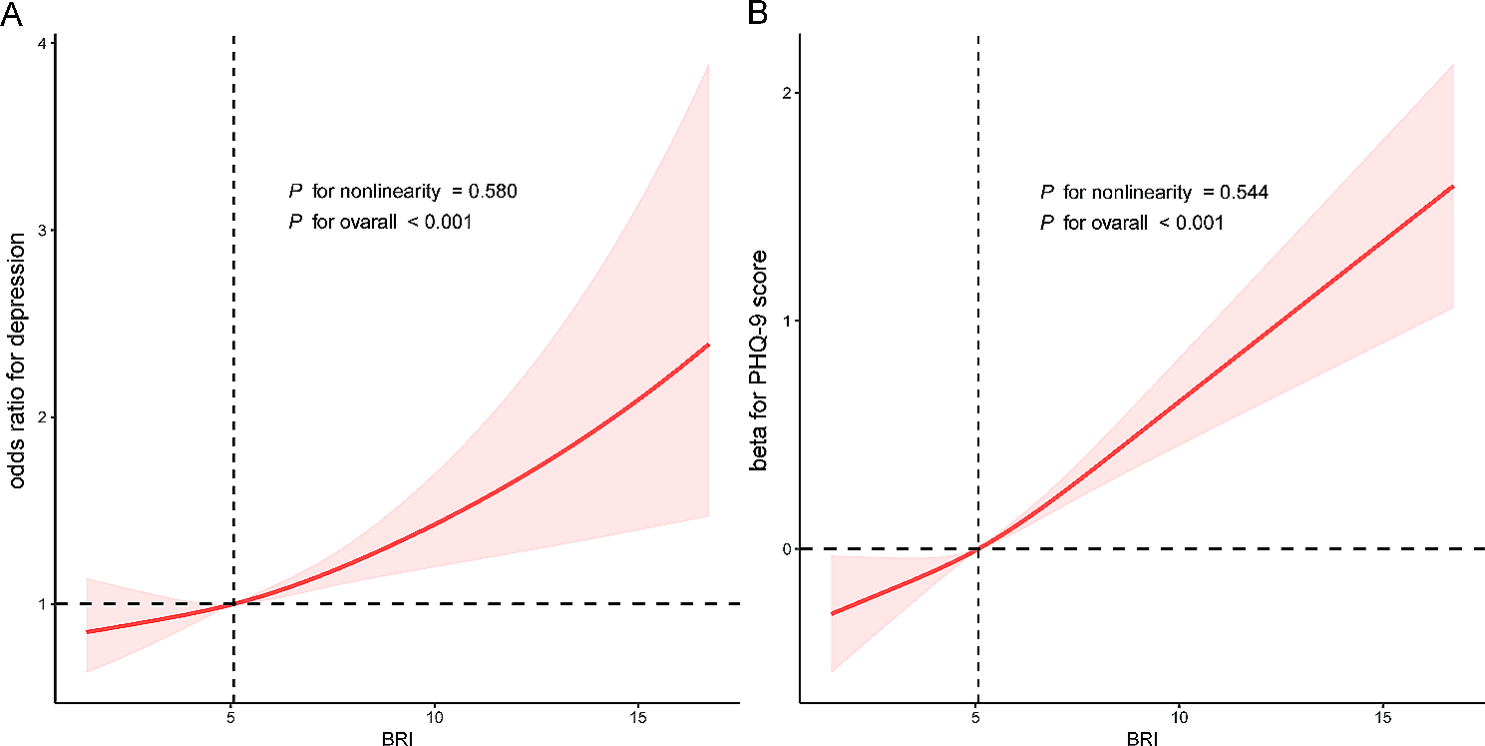
(**A**) The dose–response relationship between BRI and PHQ-9 score; (**B**) The dose–response relationship between BRI and depression. The associations were adjusted for age, sex, race, education level, marital status, poverty income ratio, smoking status, alcohol status, diabetes status, cardiovascular disease status, chronic kidney disease status, cancer status and antidepressant use

As shown in Table 2, the BRI was positively correlated with the PHQ-9 score in both the crude model (β coefficients: 0.20, 95% CI: 0.16–0.23) and the fully adjusted model (β coefficients: 0.12, 95% CI: 0.08–0.16). A similar correlation between the BRI and depression was also observed. After adjusting for all covariates, the likelihood of depression increased by 7.0% for each unit increase in the BRI (OR: 1.07, 95% CI: 1.04–1.10). Furthermore, this study revealed that individuals in the highest BRI (Q4) group had an OR of 1.42 for depression (95% CI: 1.12–1.82) in comparison with participants in the lowest BRI (Q1) group. RCS analyses indicated a linear association between the BRI and depression, as well as between the BRI and PHQ-9 score (Fig. 2).

**Table 2:** Associations between body roundness index and depression

BRI	PHQ-9 score	Depression
	[β (95%CI)]	[OR (95%CI)]
Crude model (model 1)		
Continuous	0.20 (0.16, 0.23)	1.13 (1.10, 1.16)
Categories		
Quartile1	0 (ref)	1 (ref)
Quartile2	0.03 (-0.23, 0.28)	1.01 (0.74, 1.37)
Quartile3	0.26 (0.03, 0.48)	1.21 (0.95, 1.55)
Quartile4	1.15 (0.89, 1.40)	2.00 (1.62, 2.48)
*P* for trend	< 0.001	< 0.001
Minimally adjusted model (model 2)		
Continuous	0.19 (0.15, 0.23)	1.12 (1.09, 1.15)
Categories		
Quartile1	0 (ref)	1 (ref)
Quartile2	0.16 (-0.09, 0.41)	1.03 (0.74, 1.42)
Quartile3	0.40 (0.17, 0.63)	1.19 (0.92, 1.55)
Quartile4	1.16 (0.88, 1.43)	1.70 (1.34, 2.16)
*P* for trend	< 0.001	< 0.001
Fully adjusted model (model 3)		
Continuous	0.12 (0.08, 0.16)	1.07 (1.04, 1.10)
Categories		
Quartile1	0 (ref)	1 (ref)
Quartile2	0.10 (-0.14, 0.34)	0.99 (0.71, 1.40)
Quartile3	0.26 (0.02, 0.51)	1.12 (0.84, 1.49)
Quartile4	0.67 (0.40, 0.94)	1.42 (1.12, 1.82)
*P* for trend	< 0.001	0.001

Model 1: no covariates were adjusted

Model 2: age, sex, and race were adjusted

Model 3: age, sex, race, education level, marital status, PIR, smoking status, alcohol status, diabetes status, cardiovascular disease status, chronic kidney disease status, cancer status and antidepressant use were adjusted. BRI, body roundness index. 95%CI, 95% confidence interval

### Subgroup analysis

To investigate the association between the BRI and depression across diverse populations stratified by age, sex, the PIR, education level, and disease status (diabetes, CVD, CKD, and cancer), subgroup analyses were performed. This study revealed a significant gender interaction effect between the BRI and depression (interaction *P* value < 0.05). Among females, each one-unit increase in the BRI corresponded to a 14.0% increase in the incidence of depression (OR: 1.14, 95% CI: 1.11–1.18). However, other covariates, such as age and PIR, were not identified to have interactive effects on the association between depression and BRI (Table 3).

**Table 3:** Subgroup analysis of the association between BRI and depression

Subgroup	Depression [OR(95%CI)]	*P* for interaction
Age group		0.654
< 60	1.13 (1.10, 1.16)	
≥ 60	1.15 (1.07, 1.23)	
Sex		0.012
Male	1.05 (0.99, 1.12)	
Female	1.14 (1.11, 1.18)	
Poverty ratio		0.197
< 1.30	1.10 (1.06, 1.15)	
1.31 ~ 3.50	1.08 (1.02, 1.14)	
> 3.50	1.18 (1.08, 1.28)	
Education level		0.063
Less than high school	1.06 (0.94, 1.20)	
High school	1.08 (1.04, 1.13)	
More than high school	1.16 (1.11, 1.21)	
Cardiovascular disease		0.597
No	1.11 (1.08, 1.15)	
Yes	1.14 (1.05, 1.23)	
Diabetes		0.274
No	1.11 (1.07, 1.14)	
Yes	1.15 (1.08, 1.22)	
Chronic kidney disease		0.312
No	1.13 (1.10, 1.17)	
Yes	1.09 (1.01, 1.17)	
Cancer		0.169
No	1.14 (1.10, 1.17)	
Yes	1.06 (0.97, 1.16)	

Abbreviation: BRI, body roundness index; Patient Health Questionnaire-9, PHQ-9

Abbreviation: BRI, body roundness index; AIP, Plasma atherogenic index

### Mediation analysis

**Fig. 3:**
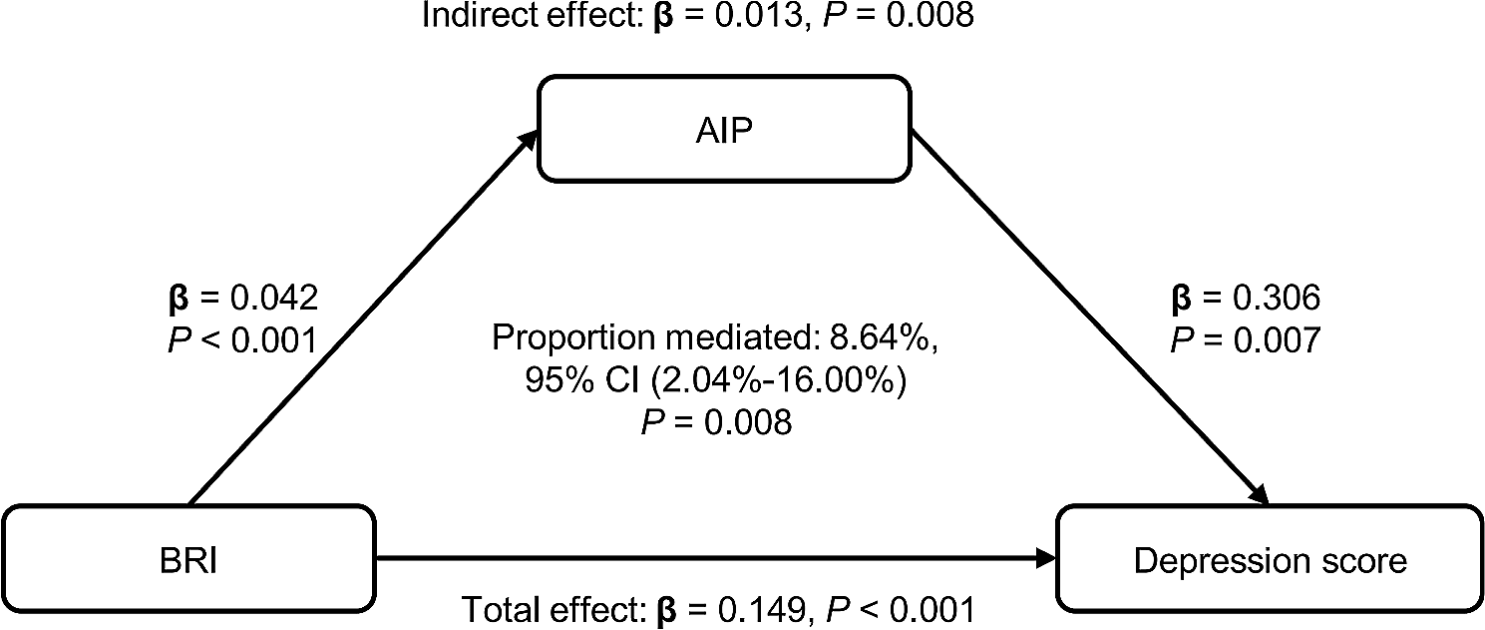
Estimated proportion of the association between BRI and depression mediated by AIP

In the mediation analysis, the BRI, the AIP, and depression were considered the independent variable, mediator variable, and dependent variable, respectively. The mediation model and paths are shown in Fig. 3. The research findings demonstrated a noteworthy correlation between the BRI and AIP (β coefficients: 0.04, 95% CI: 0.01–34.42), as well as between the AIP and depression (β coefficients: 0.31, 95% CI: 0.11–2.72). Further analysis demonstrated a noteworthy indirect impact of the BRI on depression through the AIP, with an indirect effect size of 0.013 (*P* = 0.008). It suggests that the AIP partially mediated the association between the BRI and depression, accounting for approximately 8.64% (95% CI: 2.04-16.00%) of the total effect.

## Discussion

This study revealed a positive association between BRI and depression, with a notably stronger association among females. Additionally, the mediation analysis revealed that the AIP partially mediated the association between the BRI and depression.

The current research represents one of the largest investigations to date in exploring the relationship between the BRI and depression, involving a representative cohort of 13,123 American adults from the NHANES. A previous cross-sectional study in the elderly population in China discovered a positive correlation between the BRI and depression [28], aligning with the results of this study. However, Lotfi et al.‘s study conducted among healthcare and administrative personnel in Iran did not reveal this association [29]. The difference in research findings between Lotfi’s study and ours can be explained by the following reasons. First, our study sample covered a broader range, including populations of different ages, and races. The mean age of individuals in our study is 47.51 years old, whereas Lotfi’s study reported a mean participant age of 36.6 years. Lotfi’s study involved a younger population, and age is an important covariate that may affect the association between BRI and depression. Second, this study used the PHQ-9 for depression screening, while Lotfi et al. used the HADS. However, in the general population, the PHQ-9 has shown higher levels of sensitivity and specificity, which is beneficial for identifying depression patients [30].

The interaction analysis revealed that there may be a certain interaction effect between the BRI and sex on depression, with the BRI having a greater impact on depression in females. Firstly, this may be because depression is more common among females. Another possible explanation is that sex differences lead to differences in hormone levels, consequently influencing the correlation between BRI and depression. For example, oestrogen has an impact on fat distribution [31] and the occurrence of depression [32]. Oestrogen is believed to regulate the distribution of fat, favouring the accumulation of subcutaneous fat rather than visceral fat [31]. Moreover, baseline levels and fluctuations in oestrogen are thought to increase the risk of depression in females [32], so the accumulation of visceral fat could be strongly linked to depression in females. In addition, there are other possible mechanisms that are worthy of further research.

The research findings indicate that the AIP partially mediates the correlation between visceral obesity (as determined by the BRI) and depression. This suggests that monitoring the AIP levels of patients with high BRIs is crucial. Previous researches have indicated a close relationship between lipid metabolism disorders and depression [12, 33]. Regulating the AIP, especially by increasing HDL-C levels and reducing TG levels, may help reduce the risk of depression in individuals with high BRIs.

### Strengths and limitations

The study presented several advantages. Firstly, compared to methods such as X-ray and computed tomography, which require complex diagnostic equipment or invasive assessments of visceral fat, the BRI is simpler, more convenient, easier to implement, provides greater clinical utility, and has economic feasibility [8]. Secondly, this study utilized weighted NHANES national sample data, which can better reflect the relationship between the BRI and depression in American adults. Thirdly, we thoroughly reviewed previous literature, considered and controlled for various potential confounders that might influence the relationship between the BRI and depression, and employed multivariable regression models to derive more accurate conclusions. Fourthly, this study conducted an intermediary analysis to investigate the associations between lipid-related characteristics, visceral obesity, and depression.

This study also bears limitations. Firstly, the cross-sectional design of the study precludes establishing a causal link between the BRI and depression. Secondly, the study cannot account for all potential confounding factors, such as adverse childhood experiences and personality traits. These variables are related to visceral fat [34–36] and depression [37, 38], but the NHANES database lacks relevant variable records. Thirdly, the slightly low proportion of mediation by the AIP suggests that further research may be necessary to explore the mechanisms that underlie the association between the BRI and depression. Moreover, the study found interaction effects between gender and BRI on depression, suggesting the necessity for additional investigation.

## Conclusion

The BRI was positively associated with depression, with the AIP playing a mediating role. This study provides a novel perspective on the mechanism that connects visceral obesity to depression. Managing visceral fat and monitoring AIP levels may contribute to alleviating depression.

### Electronic supplementary material

Below is the link to the electronic supplementary material.

Supplementary Material 1

## Data Availability

No datasets were generated or analysed during the current study.

## Abbreviations

BMI: Body Mass Index
WC: Waist circumference
BRI: Body Roundness Index
CVD: Cardiovascular Disease
AIP: Atherogenic Index of Plasma
NHANES: National Health and Nutrition Examination Survey
PHQ-9: Patient Health Questionnaire-9
PIR: Poverty income ratio
CKD: Chronic Kidney Disease
HDL-C: High-density lipoprotein cholesterol
TG: Triglyceride
CI: Confidence interval
OR: Odds ratio
RCS: Restricted cubic spline
