# Supplementary material for: Atherogenic Index of Plasma as a Mediator in the association between Body Roundness Index and Depression: insights from NHANES 2005–2018
Source: Lipids Health Dis. 2024 Jun 12;23:183. doi: 10.1186/s12944-024-02177-y (PMC11167922; doi:10.1186/s12944-024-02177-y)
Supplement: Supplementary file 1 — Supplementary Material 1 [file 12944_2024_2177_MOESM1_ESM.docx]

**Table S1. Definition and inclusion for the covariates**

Smoking status: Smoking status was recorded as never smoker, former smoker, or current smoker.

Diabetes: (1) confirmed diagnosis by a physician, (2) fasting blood glucose level ≥ 7.00 mmol/L, (3) glycated hemoglobin (HbA1c) level > 6.5%, (4) random blood glucose level ≥ 11.10 mmol/L, (5) two-hour oral glucose tolerance test (OGTT) level ≥ 11.10 mmol/L, (6) current use of diabetes medication or insulin.

Cardiovascular disease: Information on CVD was evaluated using verified self-administered questionnaires. Self-reported histories of CVD including coronary heart disease, angina, congestive heart failure, heart attack and hypertension.

Chronic kidney disease: The CKD diagnostic criteria follow the KDIGO 2021 Clinical Practice Guideline for the Management of Glomerular Diseases.

Cancer status and antidepressant use status: Information on cancer status and antidepressant use status was evaluated using verified self-administered questionnaires.
